# Malaria transmission heterogeneity in different eco-epidemiological areas of western Kenya: a region-wide observational and risk classification study for adaptive intervention planning

**DOI:** 10.1186/s12936-024-04903-4

**Published:** 2024-03-12

**Authors:** Guofa Zhou, John Githure, Ming-Chieh Lee, Daibin Zhong, Xiaoming Wang, Harrysone Atieli, Andrew K. Githeko, James Kazura, Guiyun Yan

**Affiliations:** 1 Program in Public Health, University of California, Irvine, CA USA; 2 Sub-Saharan International Center of Excellence for Malaria Research, Tom Mboya University, Homa Bay, Kenya; 3 Centre for Global Health Research, Kenya Medical Research Institute, Kisumu, Kenya; 4 Center for Global Health and Diseases, School of Medicine, Case Western Reserve University, Cleveland, OH USA

## Abstract

**Background:**

Understanding of malaria ecology is a prerequisite for designing locally adapted control strategies in resource-limited settings. The aim of this study was to utilize the spatial heterogeneity in malaria transmission for the designing of adaptive interventions.

**Methods:**

Field collections of clinical malaria incidence, asymptomatic *Plasmodium* infection, and malaria vector data were conducted from 108 randomly selected clusters which covered different landscape settings including irrigated farming, seasonal flooding area, lowland dryland farming, and highlands in western Kenya. Spatial heterogeneity of malaria was analyzed and classified into different eco-epidemiological zones.

**Results:**

There was strong heterogeneity and detected hot/cold spots in clinical malaria incidence, *Plasmodium* prevalence, and vector abundance. The study area was classified into four zones based on clinical malaria incidence, parasite prevalence, vector density, and altitude. The two irrigated zones have either the highest malaria incidence, parasite prevalence, or the highest malaria vector density; the highlands have the lowest vector density and parasite prevalence; and the dryland and flooding area have the average clinical malaria incidence, parasite prevalence and vector density. Different zones have different vector species, species compositions and predominant species. Both indoor and outdoor transmission may have contributed to the malaria transmission in the area. *Anopheles gambiae *sensu stricto (*s.s.*), *Anopheles arabiensis*, *Anopheles funestus s.s.*, and *Anopheles leesoni* had similar human blood index and malaria parasite sporozoite rate.

**Conclusion:**

The multi-transmission-indicator-based eco-epidemiological zone classifications will be helpful for making decisions on locally adapted malaria interventions.

**Supplementary Information:**

The online version contains supplementary material available at 10.1186/s12936-024-04903-4.

## Background

Although it is preventable and treatable, malaria continues to be one of the greatest global public health threats, despite considerable progress made through intensive interventions in the past two decades [1]. According to the World Health Organization (WHO), in 2021 there were an estimated 247 million malaria cases in 84 malaria-endemic countries, with an estimated 619,000 deaths. About 95% of cases occurred in Africa [1]. In 2015, the WHO set the target of reducing global malaria incidence and mortality by at least 90% by 2030 [2], and many endemic African countries have set their milestones with the aim of eliminating malaria by 2030 [3–6]. However, controlling malaria requires effective strategies, and to achieve optimal cost-effectiveness the strategies must adapt to local eco-epidemiological settings [7, 8]. A better understanding of malaria biology/ecology is key to designing such intervention strategies [7–9].

Malaria is a mosquito-borne infectious tropical disease transmitted by bites from infected female *Anopheles* mosquitoes. Transmission spatial heterogeneity is one of the key epidemiological characteristics of vector-borne infectious diseases, including malaria, due to the heterogeneity of environments supporting vector development and reproduction and disease pathogen transmission [10–13]. In malaria studies, researchers have found that temperature/precipitation patterns, land use land cover, elevation, and landscape features such as valley shape, rivers, and slopes all affect the transmission and distribution of malaria vectors and *Plasmodium* parasite infections [14–18]. Many studies found that *Plasmodium* infections (both symptomatic and asymptomatic) showed aggregated patterns in certain areas (i.e., hotspots) and in many cases at the household or village level [11–13, 19, 20], suggesting the potential for focused interventions [10, 21–23]. However, targeted interventions produced mixed results [10, 21, 23]. The failure of hotspot-targeted interventions to accelerate malaria elimination is likely due to confounding factors and to the incomplete understanding of spatial transmission dynamics [20, 22, 24]. Geographical micro-variations in malaria transmission may be a universal feature [22, 25, 26]. Fine-scale, i.e., household- or village-level, risk factor analysis is useful for informing household-based intervention strategies, but it may not be suitable for regional- or national-level intervention planning and implementation [21, 22, 25, 27]. It is important to optimally utilize the spatial information collected to prioritize locally adapted interventions in resource-limited settings. The question is how to make use of household/village-level information for regional malaria control planning.

Malaria risk can be measured using several indicators, including asymptomatic *Plasmodium* infection prevalence, clinical malaria incidence, and vector density [21, 24, 28–30]. These indicators are also used for spatial heterogeneity and household/village-level risk assessments [11–13, 19, 31]. In addition to cluster/village-level heterogeneity analyses, a recent study shows that through in-depth analyses the same information can also potentially be used for regional intervention planning [32]. The selection of interventions is based on epidemiological status, but it should be adapted to the local ecological conditions that support the development and survival of malaria vectors. Previous study used *Plasmodium* infection prevalence, *Anopheles* adult density, and clinical malaria incidence from different seasons to classify study areas into different eco-epidemiological zones [32], which can potentially be used for intervention planning. However, previous study did not analyse the potential spatial heterogeneity of malaria transmission, and the study area was relatively small. An expanded study area including more diverse eco-epidemiological areas would enable researchers to draw generalized conclusions.

In this study, the study area was expanded to include areas with more diverse eco-epidemiological conditions. The spatial heterogeneity of transmission was examined based on epidemiological and entomological observations and conducted classification analyses to determine the eco-epidemiological zones. The eco-epidemiological zone classifications will be helpful for making decisions on locally adapted malaria interventions.

## Methods

### Study area

**Fig. 1:**
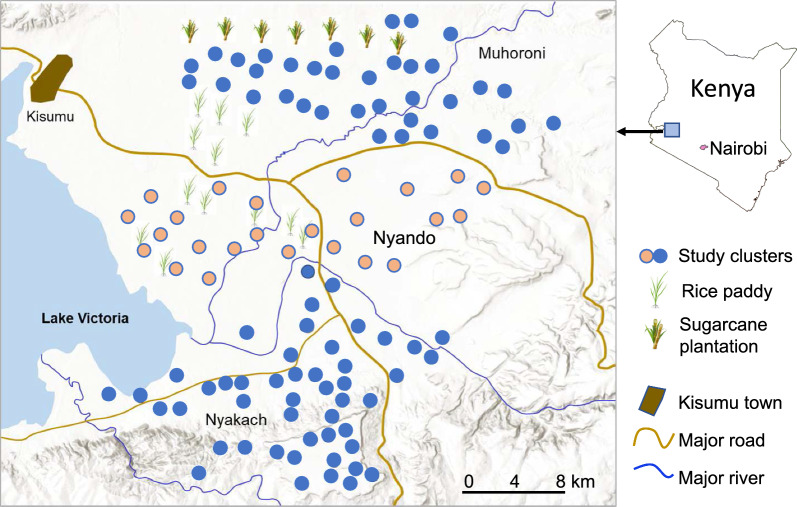
Distribution of study clusters in Muhoroni (top), Nyando (middle), and Nyakach (bottom) sub-counties, Kisumu, Kenya

The study was conducted in 108 clusters in Muhoroni (top), Nyando (middle) and Nyakach (bottom) sub-counties of Kisumu County, western Kenya (Fig. 1). The study areas cover an area of about 1,440 km^2^ and a population of about 466,000. The climatic/environmental conditions and the definition of a cluster have been described in previous studies [32, 33]. The north end of the Muhoroni study area is a large sugarcane plantation, and the central western area borders rice paddies (Fig. 1). In the south, about half of the Nyakach study area is on the Lake Victoria shore plain with swamps (elevation 1,140–1,200 m), with a sloped transition area leading to a highland plateau (elevation 1,550–1,650 m) at the southern border of the study area (Fig. 1). Nyando lays in the centre of the Kano plain along the Lake Victoria shoreline. Nyando presents a contrast in agricultural practices between its western and eastern halves, with rice fields dominating the western side and dryland maize cultivation in the flooded (during the long-rainy season) plains of the eastern region (Fig. 1). Malaria transmission in the study area is perennial with an annual peak from May to July during the long rainy season [32, 33]. Malaria vectors are *Anopheles funestus *sensu lato (*s.l*.) and *Anopheles arabiensis* in the Muhoroni area and *An. funestus*, *An. arabiensis* and *Anopheles gambiae *sensu stricto (*s.s*.) in the Nyakach area [32–34]. Malaria vector species in Nyando area is not clear. Also present are *Anopheles coustani*, a potential malaria vector in Kenya, and *Anopheles pharoensis* [32–35].

### Entomological survey

Cross-sectional indoor and outdoor mosquito surveys were conducted at all 108 clusters. Indoor-resting mosquitoes were collected using the pyrethrum spraying collection (PSC) method. Both indoor and outdoor host-seeking mosquitoes were collected using CDC light traps. In each cluster for each sampling method, 20–25 houses were randomly selected, and samplings were conducted monthly from May to September 2021. Location and altitude of sampling houses were determined using a handheld GPS. Mosquito species were identified morphologically, and female anopheline mosquitoes were classed as unfed, blood fed, half-gravid, and gravid. Specimens of *An. gambiae s.l.* and *An. funestus s.l.* were further analysed by rDNA-PCR for species identification. Mosquito density was calculated as the number of *Anopheles* females per house per night.

### Asymptomatic parasite infection prevalence

Cross-sectional *Plasmodium* parasite infection surveys were conducted from May to September 2021. The details of the sample collections have been described in previous study [32]. Briefly, about 100–120 participants were randomly selected at each cluster. On signing of the informed consent/assent (for minors younger than 18 years) forms, blood samples were collected using the standard finger-prick method. Thin and thick blood smears were prepared for laboratory microscopy examination, and filter paper blood dots were prepared for PCR detection of parasite infection status and species. Parasite prevalence was calculated as the proportion of positive samples over total samples tested based on the PCR test results.

### Active malaria case detection

Clinical malaria incidence was determined through active case detection (ACD) conducted at all clusters from April to September 2021. The details of ACD have been described in previous studies [30, 32]. Briefly, a cohort of 100–150 households, comprising about 500 residents, was selected randomly from each cluster, and all residents in the selected households were invited to participate in the study based on the inclusion and exclusion criteria. Written informed consent/assent (for minors younger than 18 years) for study participation was obtained from all consenting heads of households and from each individual who was willing to participate in the study. Participants were visited bi-weekly by a team of government trained and certified local Community Health Volunteers (CHVs) and screened for clinical malaria. Body temperature and symptoms and signs of illness were recorded on a case report form (CRF). For anybody who had malaria-like symptoms, a rapid diagnosis test (RDT) was administered on-spot. Clinical cases were referred to the nearest government-run hospital or health centre for free treatment. A clinical malaria case is defined as an individual with fever (axillary temperature of 37.5 °C or higher) and other related symptoms such as chills, severe malaise, headache, or vomiting at the time of examination or 1–2 days before the examination, together with a *Plasmodium*-positive RDT. The incidence rate was calculated as the number of cases per 1,000 people per year based on the RDT test results.

### Data statistical analyses

Spatial heterogeneity of mosquito density, parasite prevalence and clinical malaria incidence was examined using the Getis-Ord Gi* statistic of the ArcGIS Hot Spot Analysis tool (ArcGIS Pro 3.0, ESRI Inc., Redlands, CA, USA) [36]. Trend surfaces of malaria incidence, parasite prevalence and malaria vector density were produced using the completely regularized spline smoothing method of radial basis kernel function of ArcGIS. The study clusters were then classified into different zones, using hierarchical clustering method, based on the combination of all risk factors measured, including mosquito density, parasite prevalence, clinical malaria incidence, and elevation. Analysis of variance (ANOVA) was used to assess differences in parasite prevalence (with arcsine transformation), clinical malaria incidence (with logarithmic transformation), and malaria vector density among different zones. The post hoc Tukey HSD test was used for pairwise tests. The χ^2^ test was used to test the differences in *Anopheles* mosquito species composition among different zones, and differences in sporozoite rate and bloodmeal sources between indoors and outdoors and between different vector species.

## Results

### Descriptive statistics

For the ACD surveillance, 10–12 rounds of home visits were carried out, 364,176 person-visits were conducted, and 1,862 RDT-positive malaria cases were detected, with an overall annual incidence rate of 122.7 cases/1,000 people (95% CI 121.6–123.8) (Table 1).

**Table 1:** Summary of clinical malaria incidence, parasite prevalence, and vector density

Items	Numbers	Note
Number of study clusters	108	
Malaria clinical incidence		
Population enrolled (individuals)	45,738	
Rounds of home visits	5–12	Bi-weekly
Total person-visits	364,176	All ages
RDT positive cases	1,862	
Incidence rate (95% CI)	122.7 (121.6–123.8)	Cases/1,000 people/year
Parasite prevalence		
Blood samples collected	11,554	All ages
PCR detected prevalence (95% CI)	29.2% (28.3–30.1)	
*Anopheles* density		
Trap-nights sampled	5,173	All-inclusive
*Anopheles* captured	12,600	Total captures
Indoor resting density	1.0 ± 1.7	Females/house/night (± SD)
Indoor host-seeking density	1.2 ± 1.6	
Outdoor host-seeking density	1.1 ± 2.0	

For the *Plasmodium* prevalence surveys, a total of 11,554 blood samples were collected and PCR was done on 9,184 samples (Table 1). PCR detected 2,686 infected samples, a prevalence of 29.2% (95% CI 28.3–30.1). Among the PCR-positive samples, 2,541 (94.6%) were *Plasmodium falciparum*, 26 (1.0%), *Plasmodium malariae*, 34 (1.3%), *Plasmodium ovale*, 15 (0.6%) mixed *P. falciparum* and *P. ovale*, and 60 (2.2%) mixed *P. falciparum* and *P. malariae*.

For the vector surveillance, a total of 5,173 trap-nights were sampled, and 12,600 *Anopheles* mosquitoes were captured (Table 1). Among the mosquitoes captured, 9,020 (72.6%) were morphologically identified as *An. gambiae s.l*., 2,218 (17.6%) *An. funestus s.l*., 825 (6.5%) *An. coustani*, 425 (3.4%) *An. pharoensis*, and 112 (0.9%) unidentified species. PCR analyses found that among *An. gambiae s.l*., 89.5% (759/848) were *An. arabiensis* and the rest were *An. gambiae s.s*.; among *An. funestus s.l*., 86.3% (761/882) were *An. funestus s.s.* and the rest were *An. leesoni*. A total of 46,050 *Culex* and 54 *Aedes* were collected.

Although there were significant time-lagged correlations between *Anopheles* densities and parasite prevalence and clinical malaria incidence (Additional file 1: Table S1), the overall pairwise correlations between the four malaria transmission indicators, parasite prevalence, clinical malaria incidence, vector density and elevation, were not very high, with the highest correlation of 0.41 between parasite prevalence and clinical malaria incidence (Additional file 1: Table S2).

### Trend surface and hot spot analyses

**Fig. 2:**
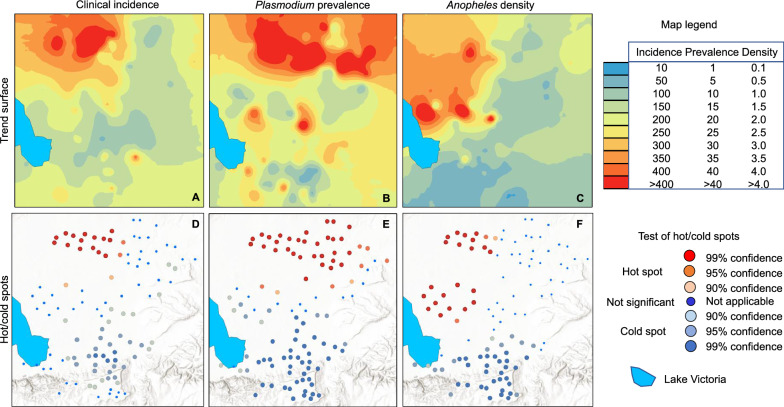
Maps of trend surface and hotspots in parasite prevalence (**A**/**D**), clinical malaria incidence (**B**/**E**), and malaria vector density (**C**/**F**) in the study area

Trend surface analysis illustrated strong spatial heterogenous distribution of clinical malaria incidence (Fig. 2A), asymptomatic parasite prevalence (Fig. 2B), and malaria vector density (Fig. 2C). Clinical incidence was very high in the northwestern section of the study area, parasite prevalence was high across the northern part plus a few sparse clusters across the study area, while vector density was high across the northwestern part of the study area (Fig. 2A–C).

Both hot and cold spots were detected in the study areas (Fig. 2D–F). A small hotspot of clinical incidence was detected in the northwestern area (Fig. 2D). For parasite prevalence, a large hotspot was detected in the north and a large cold spot in the south (Fig. 2E). For vector density, cold spots were detected in the south similar to that of prevalence, while the locations of the hot spots were different from both clinical incidence and parasite prevalence (Fig. 2F). In general, at the top of the highland plateau there was low parasite prevalence, low malaria incidence and low vector density, while the northern area surrounded by the sugarcane plantation and rice fields had high parasite prevalence, high malaria incidence and high vector density (Fig. 2).

### Malaria risk classification and characterization

**Fig. 3:**
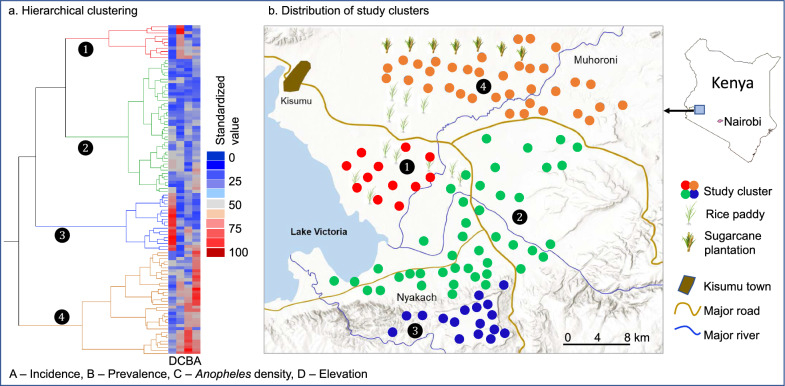
Eco-epidemiological classification of study clusters based on parasite prevalence, clinical malaria incidence, and malaria vector density. Numbers on the map corresponding to the number on the dendrogram of the hierarchical clustering on the left

**Fig. 4:**
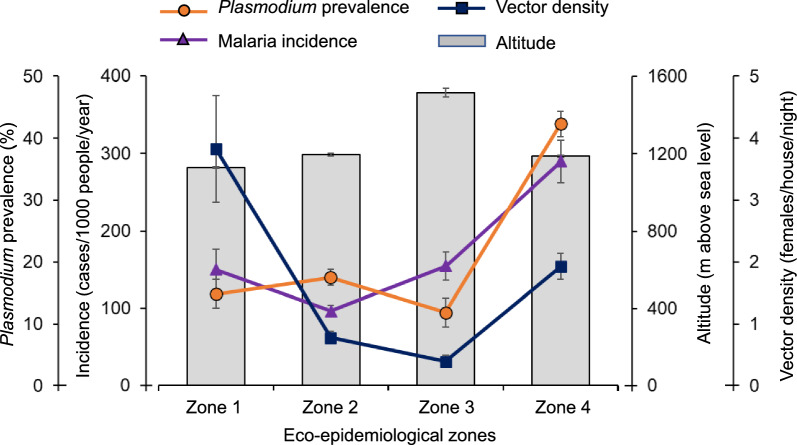
Eco-epidemiological characteristics of each zone

The study areas could be classified into four zones based on parasite prevalence, clinical malaria incidence, malaria vector density, and elevation (Fig. 3). Parasite prevalence was significantly higher in the north (42.3 ± 2.0%) (Figs. 3, 4 Zone 4) compared to the other zones (11.8–17.5%) (Tukey HSD test, P < 0.05, Figs. 3, 4). The northern zone (Zone 4 on Fig. 3) had significantly higher clinical malaria incidence (290.3 ± 27.5 cases/1,000 people/year) and Zone 2 had significantly lower clinical malaria incidence (96.5 ± 7.2 cases/1,000 people/year) (Tukey HSD test, P < 0.05, Figs. 3, 4). Zone 1 had the highest malaria vector density (3.8 ± 0.9 females/house/night) and the highland (Zone 3) had the lowest vector density (0.4 ± 0.1 females/house/night) (Tukey HSD test, P < 0.05, Figs. 3, 4). Clearly, the highland zone had the highest elevation (1514 ± 22 m above sea level) compared to the other zones (1128–1193 m a.s.l.) (Tukey HSD test, P < 0.05, Figs. 3, 4).

**Fig. 5:**
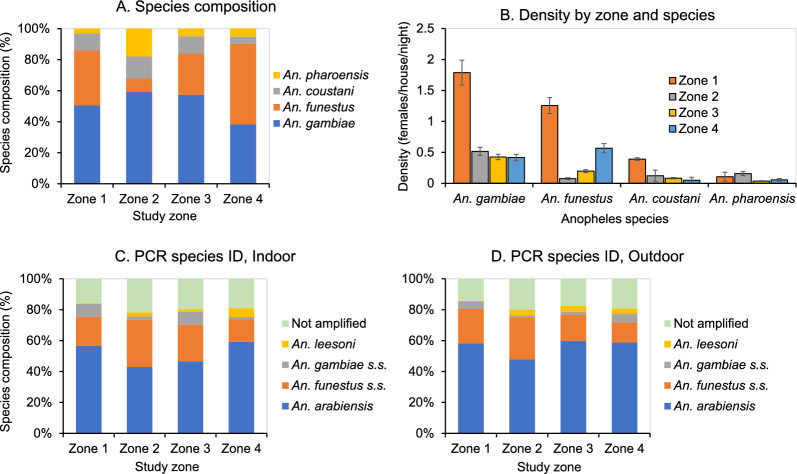
Malaria vector species composition (**A**) and abundance (**B**) in different zones, and PCR identified species compositions of indoor (**C**) and outdoor (**D**) *Anopheles gambiae *sensu lato and *Anopheles funestus s.l*

From a malaria transmission and vector control point of view, malaria vector species composition is also an important parameter. *An. gambiae s.l*. was dominant in all lowland zones while *An. funestus* was dominant in the highlands (χ^2^ = 838.09, d.f. = 9, P < 0.0001, Fig. 5A). Female density varied significantly among zones for all species (ANOVA, P < 0.05 for all). *Anopheles gambiae* and *An. funestus* had significantly higher densities in Zone 1, and the density of *An. funestus* was also high in Zone 4 (highland), while *An. coustani* had the highest density in Zone 1 (Fig. 5B). Density of *An. pharoensis* was low in all zones (Fig. 5B).

For *An. gambiae* and *An. funestus* complexes, 3,291 females were randomly selected for PCR species identifications. A total of 2,660 females have been identified to species with 1,708 (64.2%) *An. arabiensis*, 116 (4.4%) *An. gambiae s.s*., 736 (27.7) *An. funestus s.s*., and 100 (3.8%) *An. leesoni*. The species compositions were significantly different between indoor and outdoor catches (χ^2^ = 14.75, d.f. = 4, P = 0.0052, Figs. 5C, D), as well as among different zones for indoor catches (χ^2^ = 120.60, d.f. = 12, P < 0.0001, Fig. 5C) and for outdoor catches (χ^2^ = 45.73, d.f. = 12, P < 0.0001, Fig. 5D).

### Bloodmeal source and sporozoite infections

A total of 1,730 female mosquitoes were examined for bloodmeal source and sporozoite infections, of them 65 (3.8%) had sporozoite infections, 117 (6.8%) had bovine bloodmeals, 66 (3.8%) had human blood meals, and human bloodmeal index (HBI) was 0.36 (Table 2). Interestingly, sporozoite rate was significantly higher in mosquitoes collected outdoors (5.0%) compared to indoors (2.9%) (χ^2^ = 4.91, d.f. = 1, P = 0.0265, Table 2). Proportions of female mosquitoes having bovine bloodmeals was also significantly higher in mosquitoes collected outdoors (8.4%) compared to indoors (5.7%) (χ^2^ = 4.84, d.f. = 1, P = 0.0278, Table 2). Proportions of female mosquitoes having human bloodmeals was similar between mosquitoes collected outdoors (3.7%) and indoors (3.9%) (χ^2^ = 0.04, d.f. = 1, P = 0.8314, Table 2). Sporozoite infections have been detected from all four species of *An. gambiae* and *An. funestus* complexes, but sporozoite rates varied among species and between indoor and outdoor collected females, with the highest sporozoite rate of 9.1% for indoor collected *An. leesoni* and 0% for outdoor collected *An. gambiae s.s.* (χ^2^ = 3.48, d.f. = 1, P = 0.0622, Table 2). Due to the small number of sporozoite infected females, bloodmeal source and sporozoite infection were not analysed by zones. Bloodmeals and sporozoite infections in *An. coustani* and *An. pharoensis* were not examined.

**Table 2:** Blood meal source and sporozoite positivity rate of *Anopheles gambiae s.l.* and *Anopheles funestus s.l*. mosquitoes

Species	N samples	n. Pf	Bovine	Human	Not amplified	Sporozoite rate	Human blood index
Indoor							
*An. arabiensis*	490	7	27	16	36	1.4	0.37
*An. funestus s.s*	444	17	16	21	50	3.8	0.57
*An. gambiae s.s*	53	1	8	4	8	1.9	0.33
*An. leesoni*	66	6	9	0	1	9.1	0.00
Total	1053	31	60	41	95	2.9	0.41
Outdoor							
*An. arabiensis*	269	10	15	7	6	3.7	0.32
*An. funestus s.s*	317	22	27	16	34	6.9	0.37
*An. gambiae* s*.s*	36	0	3	2	1	0.0	0.40
*An. leesoni*	55	2	12	0	3	3.6	0.00
Total	677	34	57	25	44	5.0	0.30
Grand total	1730	65	117	66	139	3.8	0.36

*Pf Plasmodium falciparum*

## Discussion

Malaria transmission heterogeneity is universal due to the nature of environmental heterogeneity. For maximum effectiveness, malaria interventions should be adapted to local eco-epidemiological conditions [7, 8, 37]. The question is how to best utilize the data on transmission heterogeneity to form the optimal strategy for malaria intervention planning. Many studies have analysed malaria transmission heterogeneity, especially micro-geographical transmission hotspots. Interventions targeting high-transmission areas have yielded mixed effectiveness [21, 23, 38]. Bousema et al*.* conducted interventions in hotspots and found that the impact on parasite prevalence of interventions targeting malaria vectors and human infections was modest, transient, and restricted to the targeted hotspot areas, suggesting that a community-wide approach may be more beneficial [21]. In an earlier study by Zhou et al*.* conducted in the highlands of western Kenya at a time when vector resistance to insecticides was low, they found that indoor residual spraying in the high-transmission area (i.e., area-wide targeted intervention) significantly reduced vector density and new *Plasmodium* infections in school-aged children across the community [23]. In the study by Zhou et al., the targeted area was not a simple small hotspot but an area where most breeding habitats and *Plasmodium* infections were located [29, 39, 40]; i.e., the selection of intervention strategy was based on the eco-epidemiological settings of the area [7, 8]. For malaria control decision makers, the important question is how to determine the different eco-epidemiological settings so that different adaptive intervention strategies can be formed. In this study, based on multiple malaria risk parameters, the study areas were classified into four eco-epidemiological conditions. Since the four zones have distinct malaria transmission characteristics, the four areas may need different intervention strategies. It should be noted that in most previous studies, detection of transmission hotspots was based on a single risk indicator, in many cases using one-time-point surveillance data [21, 24, 27–29]. This study used *Plasmodium* infection prevalence (all-age inclusive), clinical malaria incidence (six months/12 rounds of active case surveillance) and malaria vector density (six-month samplings). This comprehensive surveillance data may strengthen the reliability of the classification results.

Household- and village-level heterogeneity and risk analysis is important for informing household malaria prevention strategies. However, decision-makers need regional-level eco-epidemiological analysis to form the basis for malaria control planning. For example, in the highlands, malaria vector habitats and *Plasmodium* infections are usually concentrated in the valleys [29, 31, 39, 40]. At the top of the highland plateau, habitats are usually man-made water ponds and sparsely distributed [32]; in these areas, habitat management may be an effective strategy for malaria control [23, 41, 42]. On the other hand, in the rice-growing area, although larviciding has proven to be an effective vector control strategy [43], large-scale larviciding in rice fields can be costly in terms of larvicide usage and implementation costs if larvicides are applied in multiple rounds during the rice-growing season [44], making it a less cost-effective strategy. From this point of view, this study provides useful information for vector control planning; i.e., the four zones may need different vector and malaria management strategies. For example, in the high-prevalence and high-incidence zones, an effective strategy might be enhancing diagnosis and clinical treatment to reduce the parasite reservoir, supplemented by indoor spraying with the new formulation of insecticides such as pirimiphos-methyl Actellic^®^ 300CS [35, 45, 46]. Cost-effectiveness should be considered when selecting any intervention strategy.

Malaria risk changes over time, and both malaria risk and intervention strategies may need to be reevaluated over time to adapt to the changed epidemiology [20, 22, 24]. For example, in western Kenya, supplementing LLINs with Actellic^®^ IRS has significantly reduced the malaria burden in both Migori and Homa Bay counties [35, 45, 46]. However, malaria vector density is still high in Homa Bay irrigation sites [35], and thus malaria transmission potential is still high in the area. Vector mosquito species in Homa Bay have shifted from *An. gambiae s.s*., *An. funestus* and *An. arabiensis* to *An. arabiensis* alone after several years of the enhanced Actellic^®^ IRS intervention [35, 45]. Since *An. arabiensis* is a predominantly zoophilic and exophilic species [47, 48] and IRS and LLINs work mainly indoors (although they may reduce overall mosquito density), different vector intervention strategies may be required to eliminate malaria transmission potential. Similarly, in the lake shore lowland and highland plateau parts of the study area, Actellic^®^ IRS and piperonyl butoxide LLINs (PBO-LLINs) were implemented in the study clusters in 2020 [49]. Compared with the 2019 data from the previous study [32], malaria burden and transmission have significantly reduced, indicating the effectiveness of both PBO-LLIN and LLIN + IRS interventions. Cost-effectiveness should be analysed to determine the optimal interventions.

The higher proportion of bovine (compared to human) bloodmeals and higher sporozoite infection rate from outdoor (compared to indoor) collected female mosquitoes highlighted the importance of outdoor residual transmission of malaria in western Kenya. *An. arabiensis* is an opportunistic blood sucker and rests both indoors and outdoors [50]. Studies conducted in the same area in the 1990s found that both *An. gambiae s.s*. and *An. funestus* generally fed indoors on humans preferably and rests indoors [50–53]. However, after the universal coverage of LLINs started since mid-2000s, there are amounting reports on outdoor host seeking and resting of *An. gambiae s.s.* and *An. funestus* from various places including western Kenya [33, 34, 54, 55]. In this study, about 40% bovine bloods were detected from indoor-collected *An. funestus* and higher sporozoite rate in outdoor-collected *An. funestus*, similar HBI and sporozoite rate for indoor and outdoor collected *An. gambiae s.s*., indicating the behavioural changes in both vector species in the study area, which concurs with other studies [56–58]. Outdoor transmission may have contributed a great proportion to maintaining the high parasite prevalence detected in the study areas, cost-effective control measures need to be developed and implemented to reduce the outdoor residual malaria transmission.

The major missing part of this study is seasonal surveys. Malaria transmission in the study area peaks during the long rainy season, usually from April to June. The low season is from December to February, which is usually hot and dry [32]. A cluster-randomized adaptive intervention has been implemented and is ongoing in the study area [49], which prevents us from conducting low-season surveillance. Nonetheless, both rice and sugarcane need water (irrigation) during the usually dry, hot low-transmission season, which may affect malaria transmission in the northern part of the study area. During the dry season, farms usually irrigate sugarcane and rice fields with flood irrigation utilizing the natural river water flow, which creates huge areas of *An. arabiensis* and *An. funestus* larval breeding habitats, supporting very high malaria vector density both indoors and outdoors [33–35, 50, 53]. However, irrigation agriculture is nearly null in the southern and central eastern part of the study area, so adding dry-season surveys may not change the four-zone clustering results.

## Conclusion

In conclusion, regional heterogeneity of malaria burden and transmission is universal. Examining these heterogeneities is not only important for understanding the current malaria epidemiology but also provides useful information for planning vector control strategies. Implementing interventions adapted to regional eco-epidemiological conditions may yield the robust cost-effectiveness that is especially important in resource-deficient malaria-endemic African countries.

### Supplementary Information

**Additional file 1: Table S1.** Time-lagged correlation between Anopheles density and parasite prevalence and clinical malaria incidence. **Table S2.** Pairwise correlation between mean Anopheles density, parasite prevalence, clinical malaria incidence, and elevation. Critical value of correlation at significant level of 0.05 is 0.188.

## Data Availability

Aggregated data at cluster level is available to public and can be obtained from the correspondence author.
