# Supplementary material for: Malaria transmission heterogeneity in different eco-epidemiological areas of western Kenya: a region-wide observational and risk classification study for adaptive intervention planning
Source: Malar J. 2024 Mar 12;23:74. doi: 10.1186/s12936-024-04903-4 (PMC10935946; doi:10.1186/s12936-024-04903-4)
Supplement: Supplementary file 1 — Additional file 1: Table S1. Time-lagged correlation between Anopheles density and parasite prevalence and clinical malaria incidence. Table S2. Pairwise correlation between mean Anopheles density, parasite prevalence, clinical malaria incidence, and elevation. Critical value of correlation at significant level of 0.05 is 0.188. [file 12936_2024_4903_MOESM1_ESM.docx]

Additional information

Table S1. Time-lagged correlation between Anopheles density and parasite prevalence and clinical malaria incidence.

| Indicator |  | Anopheles density by month | | | |
| --- | --- | --- | --- | --- | --- |
|  | *Month* | *May* | *June* | *July* | *August* |
| Prevalence |  | 0.290 | 0.009 | 0.191 | 0.375 |
| Incidence | May | -0.215 |  |  |  |
|  | June | -0.215 | -0.274 |  |  |
|  | July | -0.314 | -0.113 | 0.132 |  |
|  | August | -0.135 | 0.016 | 0.212 | 0.173 |
|  | September | -0.199 | -0.146 | -0.130 | -0.137 |

Table S2. Pairwise correlation between mean Anopheles density, parasite prevalence, clinical malaria incidence, and elevation. Critical value of correlation at significant level of 0.05 is 0.188.

|  | *Prevalence* | *Incidence* | *Density* | *Elevation* |
| --- | --- | --- | --- | --- |
| Prevalence | 1 |  |  |  |
| Incidence | 0.410 | 1 |  |  |
| Density | 0.154 | 0.165 | 1 |  |
| Elevation | -0.280 | -0.009 | -0.328 | 1 |
